# Assessment of current biomarkers and interventions to identify and treat women at risk of preterm birth

**DOI:** 10.3389/fmed.2024.1414428

**Published:** 2024-07-26

**Authors:** Michael G. Gravett, Ramkumar Menon, Rachel M. Tribe, Natasha L. Hezelgrave, Marian Kacerovsky, Priya Soma-Pillay, Bo Jacobsson, Thomas F. McElrath

**Affiliations:** ^1^Department of Obstetrics and Gynecology and of Global Health, University of Washington, Seattle, WA, United States; ^2^Department of Obstetrics and Gynecology, The University of Texas Medical Branch at Galveston, Galveston, TX, United States; ^3^Department of Women and Children's Health, Faculty of Life Sciences and Medicine, School of Life Course Sciences, St Thomas' Hospital Campus, King's College London, London, United Kingdom; ^4^Department of Women and Children’s Health, Faculty of Life Sciences and Medicine, School of Life Course Sciences, King’s College London, London, United Kingdom; ^5^Biomedical Research Center, University Hospital Hradec Kralove, Hradec Kralove, Czechia; ^6^Department of Obstetrics and Gynecology, Faculty of Medicine Hradec Kralove, Charles University in Prague, Hradec Kralove, Czechia; ^7^Department of Obstetrics and Gynaecology, The University of Pretoria School of Medicine, Pretoria, South Africa; ^8^Department of Obstetrics and Gynecology, Sahlgrenska Academy, Gothenburg University, Gothenburg, Sweden; ^9^Department of Genetics and Bioinformatics, Domain of Health Data and Digitalization, Norwegian Institute of Public Health, Oslo, Norway; ^10^Department of Obstetrics and Gynecology, Brigham and Women's Hospital and Harvard Medical School, Boston, MA, United States

**Keywords:** preterm birth, biomarkers, screening tools, interventions, limitations

## Abstract

Preterm birth remains an important global problem, and an important contributor to under-5 mortality. Reducing spontaneous preterm birth rates at the global level will require the early identification of patients at risk of preterm delivery in order to allow the initiation of appropriate prophylactic management strategies. Ideally these strategies target the underlying pathophysiologic causes of preterm labor. Prevention, however, becomes problematic as the causes of preterm birth are multifactorial and vary by gestational age, ethnicity, and social context. Unfortunately, current screening and diagnostic tests are non-specific, with only moderate clinical risk prediction, relying on the detection of downstream markers of the common end-stage pathway rather than identifying upstream pathway-specific pathophysiology that would help the provider initiate targeted interventions. As a result, the available management options (including cervical cerclage and vaginal progesterone) are used empirically with, at best, ambiguous results in clinical trials. Furthermore, the available screening tests have only modest clinical risk prediction, and fail to identify most patients who will have a preterm birth. Clearly defining preterm birth phenotypes and the biologic pathways leading to preterm birth is key to providing targeted, biomolecular pathway-specific interventions, ideally initiated in early pregnancy Pathway specific biomarker discovery, together with management strategies based on early, mid-, and-late trimester specific markers is integral to this process, which must be addressed in a systematic way through rigorously planned biomarker trials.

## Introduction

The concept of “personalized medicine” as a practice model that separates patients into different treatment categories based on prior characteristics or testing outcomes while slowly gaining sway in the practice of medicine remains uncommon in obstetrical practice (1). In principle, a patient’s level of risk for adverse outcomes during pregnancy would be assessed early in pregnancy and prenatal care would be adjusted accordingly. One of the impediments to adoption of personalized medicine in obstetrics has been confusion regarding the therapeutic or prophylactic interventions that could be offered if a patient was identified to be at increased risk. Another impediment has been the failure to recognize preterm birth as multifactorial with many causes risk factors and biologic pathways (2–4). This review will examine both the obstacles to the development of meaningful biomarkers for preterm birth then go on to suggest contemporary interventions that could be utilized to treat patients found to be at increased risk.

**Figure 1:**
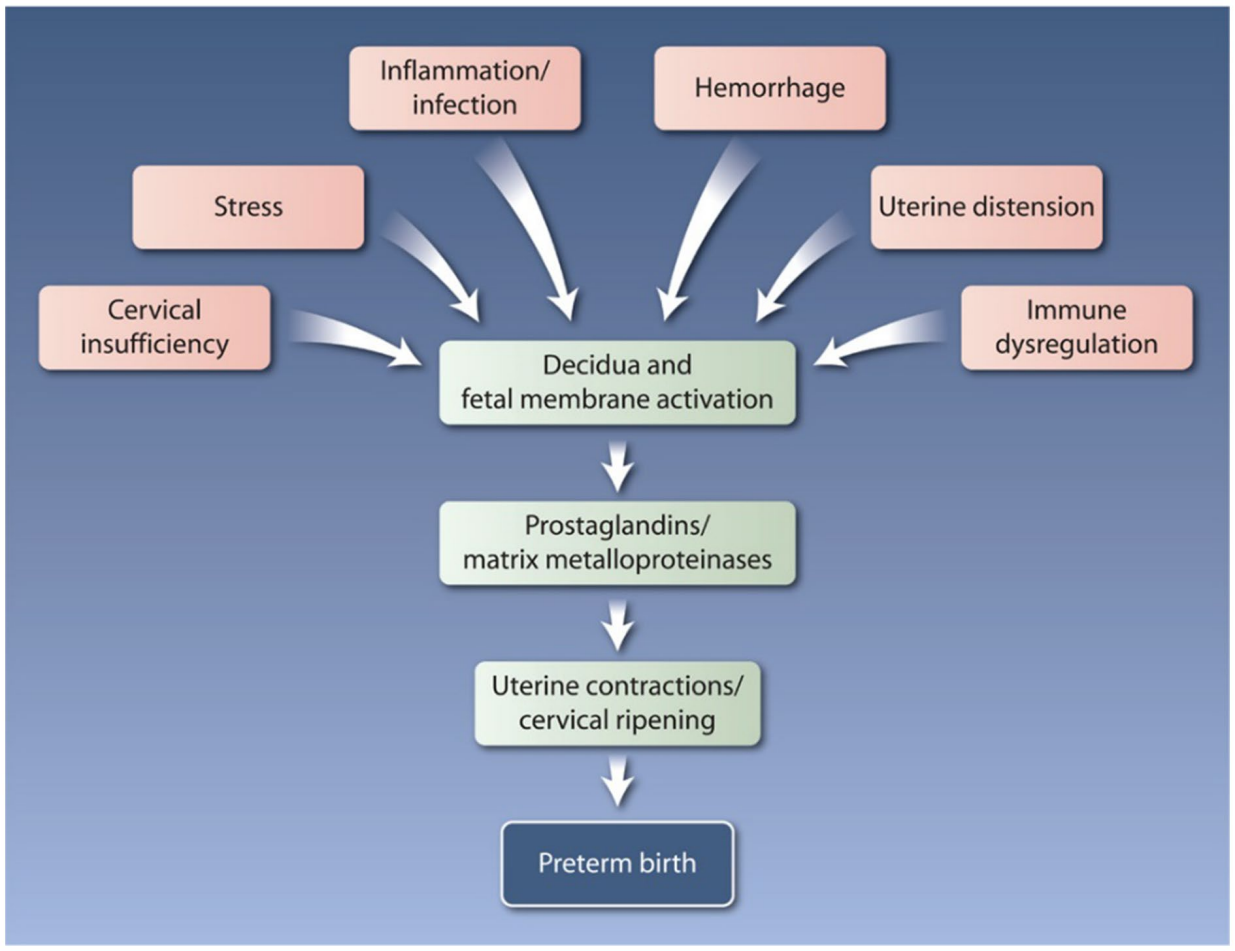
The pathways to preterm labor and preterm birth are multifactorial and complex. Multiple molecular mechanisms are influenced by a variety of risk factors, including genetic, epigenetic, biological, behavioral, social, clinical, and environmental influences. Reprinted with permission from Rubens et al. (3); © 2024 American Association for the Advancement of Science.

The complexities of the etiology of preterm birth create a major clinical diagnostic and management dilemma (1). Systematic reviews and meta-analyses on preterm birth biomarkers and clinical intervention trials have reported the need for the predictive marker(s) or approach that can successfully reduce preterm birth risk at a global level. The lack of conclusive evidence from both biomarker and intervention trials implies that preterm labor is a syndrome with multiple underlying etiologies. Heterogeneities in studying the etiology of preterm birth start with something as simple as defining the phenotype (e.g., spontaneous vs. indicated) and gestational age (very early, early, late, and very late onsets) (2). The complex interaction between various factors, e.g., epistatic interactions (gene–gene) and gene–environment (e.g., bacterial vaginosis, periodontal infection, parasitic infections, environmental exposures, and behavioral), can add more complexities to various studies’ heterogeneity as they can alter pathways, biomarkers, and gestational age at the outcome. Addressing preterm birth risk stratification and management strategies must consider tackling these sources of heterogeneity (Figure 1).

Risk scoring systems based on personal/epidemiologic risk factors have traditionally performed poorly. Presently, the most often used predictive characteristic is previous obstetric history; an indicator that precludes those in their first pregnancies while, at best, yielding a low positive predictive value. Other presently available methods such as cervical length screening and the use of qualitative fetal fibronectin (fFN) fail to identify 80% of patients who will have a preterm birth (5, 6). Recently, a blinded study in Sweden found a sensitivity of 38% and a positive predictive value of 3.6% for preterm birth with a cervical length of <25 mm at 18–20 weeks (7). Further, a recent study of 4.1 million births in high income countries revealed that no biological explanation could be found in 2/3 of all preterm births and that most patients with preterm birth had no traditionally recognized risk factors (8). It is important to recognize that not all preterm births can be predicted not anticipated. Sudden catastrophic events (e.g., placental abruption or hemorrhage from placenta previa) cannot be anticipated and many preterm births are medically indicated for maternal or fetal well-being. This review focuses on patients with known risk factors or asymptomatic screening.

**Figure 2:**
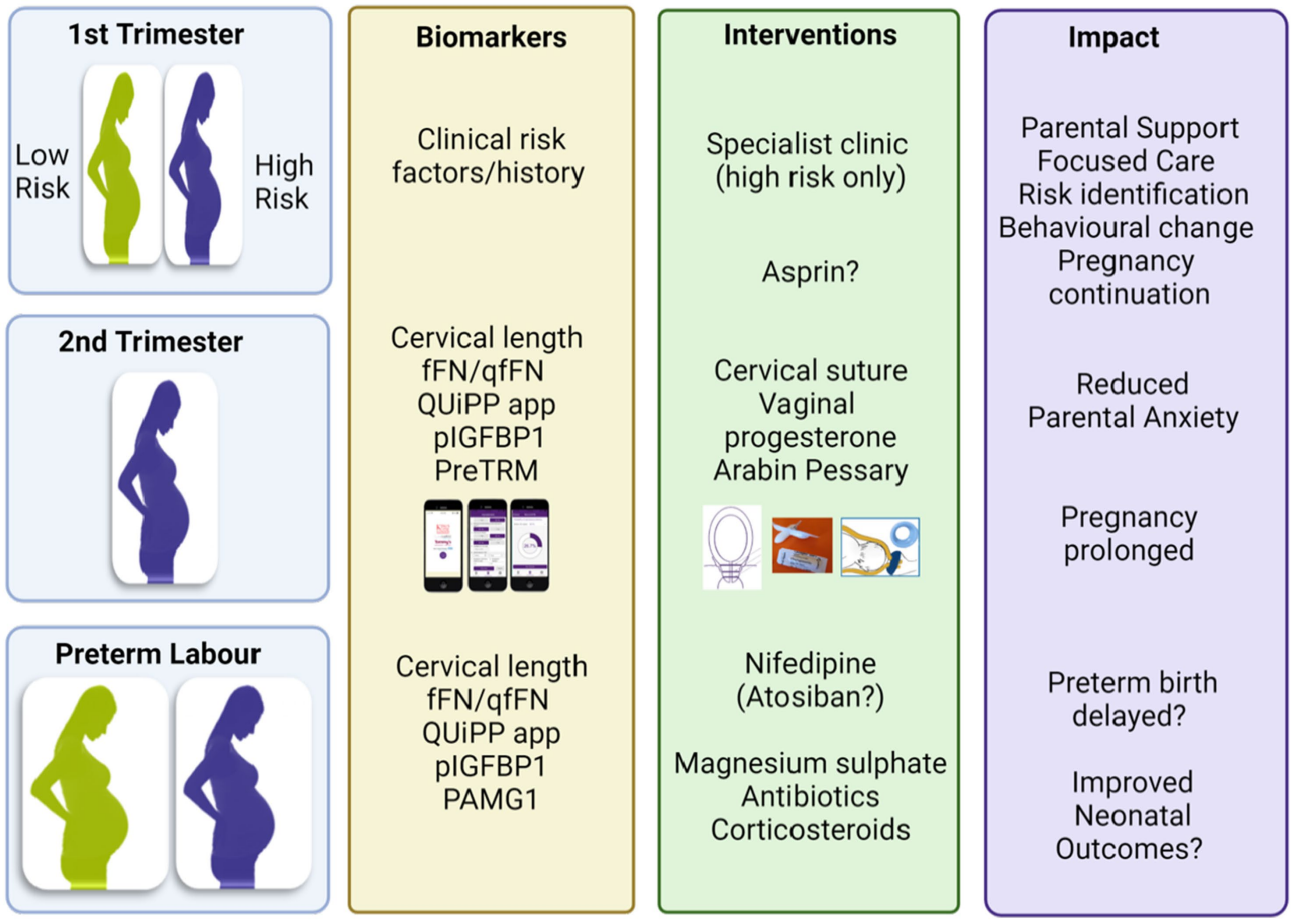
Potential biomarkers to identify women at risk of preterm birth and targeted interventions. Provided by Rachel M Tribe. fFN, fetal fibronectin; qfFN qualitative fFN; pIGFBP1, phosphorylated insuline-like growth factor binding protein 1, PAMG1, placental alpha microglobulin-1; PreTRM, PreTRM^®^.

Currently available risk factor and screening strategies include:

1. *Clinical risk factors*. As noted, the history of prior preterm birth is one of the most predictive risk factors for preterm birth. Previous preterm birth, stillbirth, or late miscarriage is associated with a as much as a 32% chance of recurrent preterm birth (9–12). The utility of this marker does not extend to patients on their first pregnancy. For the primiparous patient, there are several classic clinical risk factors associated with spontaneous preterm birth that apply to primiparous patients. These include age, ethnicity, body mass index or nutritional state, smoking, and history of bacterial vaginosis or urinary tract infections (13). However, in most clinical settings these are not used for formal risk stratification. Reports of domestic violence can trigger referrals to other types of support in pregnancy and is itself a risk for spontaneous preterm delivery (14, 15). While relatively uncommon, patients with Mullerian anomalies should be followed for risk of spontaneous preterm delivery. Women who have had prior cervical interventions for cervical intraepithelial neoplasia such as local ablative therapy or excisional methods, particularly cone biopsy, are also considered at a slightly elevated risk of spontaneous preterm birth, dependent on the degree of cervical tissue damage/loss. Unfortunately, these clinical risk factors only identify a small portion of women at risk (16). Again, there is still a great need for reliable biomarkers that can be used in low-risk and nulliparous populations that would allow early targeted intervention (Figure 2).
2. *Current tools for the assessment of biophysical risk*. Transvaginal ultrasound to measure cervical length in the mid-trimester is widely utilized to assess the risk of spontaneous preterm birth. Typically, a cervical length of less than <25 mm indicates increased risk and need for enhanced monitoring and intervention (17, 18). However, as noted above, cervical length alone detects only a minority of patients who will subsequently deliver preterm. Celik and colleagues sought to improve on this by developing an algorithm based on maternal characteristics and cervical length measurement combined with obstetric history for first trimester screening (19). However, a recent meta-analysis by Berghella and colleagues found no significant benefit for routine cervical length assessment in asymptomatic singletons with no other risk factors for preterm birth (6). Other approaches focused on the biophysics of the cervix which have been explored include cervical elastography [see (20) for review], but this has not been widely adopted in practice.
3. *Current biochemical tools*. Compared to other clinical conditions there are a lack of good biochemical based prediction tools for spontaneous preterm birth. Currently, there are only four commercially available tests (Table 1):

- PreTRM™, which is second trimester blood test (taken within an 18 and 20^+6^ weeks of gestation window). This test measures the log insulin binding protein 4 and sex hormone binding globulin ratio (IBP4/SHBG) by proteomics performed in a central laboratory. Its use has been clinically validated, with receiver operator curve characteristics (area under the curve 0.75) and has been modeled to be cost effective in treating and reducing preterm birth (21, 25, 26). This test is currently only available in the United States.
- Quantitative fFN, is a vaginal fluid swab-based test that detects fFN concentrations, a glycoprotein secreted from the decidua -chorion interface. It is a mid-trimester test that provides quantitative data of fFN concentrations that can guide clinical management of both high risk (based on clinical history) asymptomatic women and symptomatic women who have threatened preterm labor (27–29).
- Actim^®^ Partus is a less well validated cervical swab test based on the detection of a phosphorylated form of insulin-like growth factor binding protein-1 (phIGFBP-1) which has a high negative predictive value from 22 weeks of pregnancy. It has also been used for the prediction of women in threatened preterm labor (also in combination with cervical length measurement) (24) and in asymptomatic high-risk women (30, 31). However, a recent systematic review and meta-analysis by Conde-Agudelo and Romero indicated that the cervical phIGFBP-1 test has a low predictive accuracy for preterm birth at <34 and < 37 weeks of gestation in both asymptomatic and symptomatic women and for delivery within 7 and 14 days of testing among symptomatic women (31).
- PartoSure is a bedside test that detects placental alpha microglobulin-1 (PAMG1) in cervical-vaginal secretions using a vaginal swab and is reported to predict the risk of preterm birth in less than 7 days in singleton pregnancies where symptomatic women with threatened preterm labor (23).
  4. *Combined risk assessment tools*. While cervical length and fFN have been widely utilized, alone or in combination, these tests fail to identify the majority of asymptomatic patients who will ultimately have a preterm birth. In a prospective cohort study of nulliparous singleton pregnancies, only 8% of patients delivering preterm had a sonographic short cervix at 16–22 weeks, and only 7% of patients delivering preterm had a positive fFN at 16–22 weeks of gestation (22). More recently, the combination of clinical history, quantitative fFN and/or cervical length measurement (the QuiPP app) has been validated as a smartphone tool to identify patients at risk of preterm birth. Further, it has been validated in both asymptomatic high-risk patients and in symptomatic patients with threatened preterm labor (32, 33). The QuiPP app provides a calculation of the risk of preterm delivery at <30, <34, and <37 weeks or within 1, 2, and 4 weeks of testing in singleton and twin pregnancies and is approved for use in the UK, Europe, and Australia. It uses quantitative fFN and cervical length data as continuous variables and provides indications of risk of delivery at <30, <34, and <37 weeks or within 1, 2, and 4 weeks of testing, although it can also be used with cervical length and maternal history alone, independently of quantitative fFN (33).
  5. *Placental pathology*. Examination of the placental (gross examination and histopathology) is essential in appropriate risk stratification and targeted intervention in the prevention of recurrent preterm birth (34–37). For example, a recent review of 538 placentae revealed that maternal and fetal inflammation were the most important causes of extreme PTB whereas early PTB was associated with placental malperfusion, and late PTB was more frequently associated with hypoxia-related placental lesions (34). This may facilitate pathway specific targeted treatment strategies (e.g., early recognition of genital tract infections to prevent very extreme PTB or low dose aspirin to prevent early PTB). Future trials based upon previous placental therapy are warranted.

**Table 1:** Comparison of currently available tests used in screening asymptomatic women and to aid in prediction of preterm delivery for women with signs and symptoms of preterm labor.

	Cervical length	qfFN	qfFN and cervical length	IBP4/SHBG (PreTRM)	Phosphorylated IGFBP-1 (Actim Partus)
Test characteristics					
Primary intended use	Asymptomatic and symptomatic	Asymptomatic and symptomatic	Asymptomatic and symptomatic	Asymptomatic	Asymptomatic and symptomatic PTL
Specimen	Transvaginal ultrasound (TVUS)	Cervical-vaginal Swab	Cervical-vaginal swab and TVUS	Maternal blood	Cervical swab
Gestational age at testing	Asymptomatic: 20–24 weeks Symptomatic: < 35 weeks	24–35 weeks when symptoms occur		18–21 weeks	24–35 weeks
Performance					
Asymptomatic screening	++	+	++	+++	+
Symptomatic diagnosis	+++	+++	+++	N/A	++
Detection sensitivity for preterm birth in asymptomatic women	38% (9)	46% delivery <34 weeks (21)	Asymptomatic: AUC 0.77 for delivery <37 weeks (22) Symptomatic: 72% (23)	75% (19)	38% for delivery <37 weeks (24)
Clinical value	Classifier as elevated risk of preterm delivery	High negative predictive value of not delivering within 14 days	Used in conjunction with App algorithms	Targeted intervention prior to PTL	Potentially useful in symptomatic women

Performance characteristics are qualitatively based upon review of literature as modest (+), good (++), or very good (+++).

Investigational research has included exploring the relationships among the vaginal microbiome (38), vaginal metabolites (39), circulating extracellular vesicles (EV) and their content (40, 41), EV proteome (42), EV miRNA (43), cervical-vaginal metabolites (44), and the maternal plasma proteome (45) with preterm birth, but have not been commercialized or clinically validated in large studies and are not widely available. The most promising is cell-free RNA in maternal blood (46, 47). Second trimester cell-free RNA profiles in maternal plasma have been shown to have potential in identifying women at risk of a future spontaneous preterm birth and mid-trimester loss. The cell-free RNAs identified may also serve to provide insight into the mechanisms as they relate to collagen and extracellular matrix as well as metabolic and growth factors pathways for the earlier losses.

## The ideal test

While no single test will predict all preterm births, attributes of an ideal test to identify women at risk of preterm birth would include:

- High sensitivity and specificity and a high positive likelihood ratio.
- Provide for point-of-care testing.
- Validation in a variety of settings recognizing differences in geographic locale and ethnicity.
- Provide biologic pathway specificity to allow for pathway-specific targeted intervention.
- Relevant to low-and-middle income countries (LMIC) or resource challenged setting within high income countries.

Point-of-care testing (POC) testing is especially relevant to LMICs, where the burden of preterm birth is greatest. Sub-Saharan Africa and south Asia account for 60% of PTB globally, and preterm birth is now the leading cause of under-5 mortality in the world (48). Access to POC diagnostics has the potential to alleviate diagnostic challenges and delays associated with laboratory-based methods in LMICs. The ideal biomarker in these setting should fulfill the ASSURED criteria first proposed by the WHO [UNICEF/UNDP/World Bank/WHO Special Program] in 2003—Affordable, Sensitive, Specific, User-friendly, Robust and rapid, Equipment-free, and Deliverable (49, 50). The ASSURED embodies three key characteristics: accuracy, accessibility and affordability. High sensitivity is especially important for POC screening tests in LMIC to ensure that all true and suspect cases are managed or triaged to appropriate health care facilities. Point of care diagnostics fulfilling the ASSURED criteria have been demonstrated to reduce the burden of disease in several infections and communicable diseases in low resource settings (51). The increasing access to Bluetooth or wifi connectivity in LMIC now provides the opportunity to link ASSURED diagnostics to health care systems and referral centers for appropriate management or triage advise and ASSURED has been updated to REASSURED (Real-time connectivity, Ease of sample collection, Affordable, Sensitive, Specific, User-friendly, Robust and rapid, Equipment-free, and Deliverable) (51). Pregnant women living in an LMIC setting face several health system barriers such as long distances needed to access health services, poor referral systems, and unavailability of care and treatment at facility-based sites. Appropriate biochemical screening tools that fulfill the REASSURED criteria are urgently needed to reduce not only the burden of PTB but also of other adverse pregnancy outcomes (e.g., preeclampsia) in LMIC. Universal screening using facility-based tools such as cervical length measurement is done most frequently in clinics, hospitals, and other health care facilities are unlikely to be successful in LMICs.

Consideration must also be given appropriate interpretation of the result by the provider and to the appropriate communication of testing results to the patient and to the family. One initial approach would be to refrain from the use of the term “test” and instead to correctly refer call this assessment a “screening test.” The results of a putative screening test for the risk of spontaneous preterm birth needs to be communicated in a fashion that cannot inadvertently be understood as diagnostic. For example, most would not consider a high prostate specific antigen as a diagnosis of cancer. The nature of the test is understood to indicate a stratification of risk rather than diagnosis. That risk then needs to be met by therapy, behavior modification or additional testing.

One approach would be similar to approaches used in genetic screening tests used in prenatal diagnosis, during which results are presented as a relative risk of an outcome compared to the general population. More recently, the Likelihood Ratio (LR) has gained popularity (52, 53). The Likelihood Ratio (derived in from the sensitivity and specificity and calculated as sensitivity/1-specificity) computes the likelihood of an outcome among patients with a positive test (true positive) compared to those with a screen positive test that do not have the outcome (false positive). For example, in a hypothetical study, if 30% of patients who have preterm birth were screen positive and 10% of patients who are screen positive but do not have preterm birth, the LR would be 3. That is, those who have PTB are 3 times more likely to have a positive test. This is a tool to reinforce clinical judgment and can easily be communicated to the patient without suggesting the screening test is a diagnostic test. Checklists could also be employed to avoid the inadvertent omission of important topics for discussion in what is likely to be an emotionally charged encounter (54). The use of pictorial representations or graphic qualifications of risk has been much more effective than the communication of numbers, rates, or percentages (55, 56). The developers of the QuiPP app noted above found this approach to be highly effective in the communication of exactly this type of information. The risk could also be graded into “strata” or “levels,” analogous to red, yellow, and green traffic signals indicating high, medium, or low risk of preterm birth. Finally, patient advocacy organizations staffed by former patients and complication survivors exist in both the physical and virtual world and are highly effective at optimizing messaging around care and communications with patients.^1^ Simply asking a survivor’s group how they would prefer to have these results communicated would not only signal empathy but likely be highly effective.

## Potential points of intervention

While innovative technologies in pregnancy risk assessment have identified several putative predictive biomarkers for preterm birth, all suffer from a low positive predictive value, although most have a very high negative predictive value and thus could be used to as a “rule-out” test. This dilemma has led to a search for novel biomarkers with better positive prediction values early in gestation, prior to clinical presentation, that would allow for the potential modification of care in a fashion that would mitigate the risk of preterm birth. While several initiatives exist to identify appropriate biomarkers (noted above), the question then becomes one of examining the available therapies and interventions might reasonably be offered in a personalized medicine manner for each patient.

**Figure 3:**
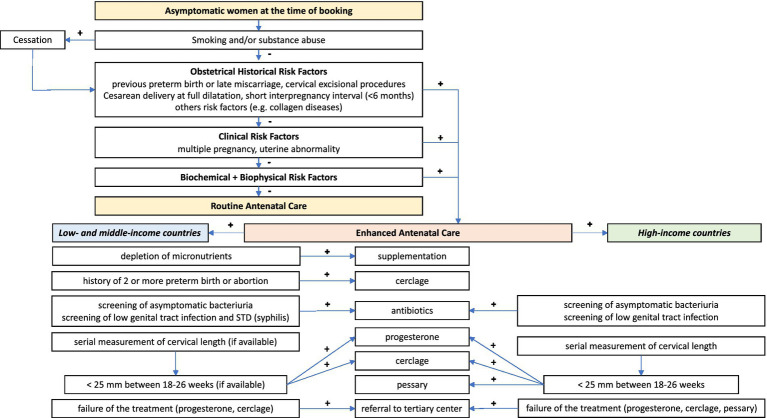
Suggested screening algorithm and possible treatment strategies for women identified as at-risk for preterm birth (singleton pregnancies). Provided by Marian Kacerovsky and Rachel M Tribe.

Current interventions to prevent preterm birth may be divided into primary, secondary, and tertiary interventions. Primary interventions are directed to all women before or during pregnancy to reduce risk (e.g., smoking cessation), secondary interventions are aimed at eliminating or reducing risk in women with known risk factors or identified as at risk), or tertiary (initiated after the parturition process has begun to prevent preterm delivery or improve outcomes for preterm infants (57). This review will focus on secondary interventions targeted at women at risk, either by preexisting risk factors or by biomarkers that identify women at risk of preterm birth (summarized in Figure 3).

## Antenatal care

Identifying patients at risk of spontaneous preterm birth would allow patients to be directed to prematurity prevention clinics and centers. Such clinics are increasingly common in tertiary referral centers in high-income-countries. In these clinics, while local protocols may vary from center to center, there are some common themes. Studies have demonstrated that such individualized care for high-risk women both identifies more patients that may benefit from medical intervention (i.e., progesterone) and reduces preterm birth (58–60).

## Current targeted interventions

Existing therapies already utilized include treatment with progesterone, cervical cerclage, pessary, and low dose aspirin. The utility of these interventions has recently been summarized in a systematic review and meta-analysis by Wennerholm et al. (61). Vaginal progesterone has been demonstrated to reduce preterm births among selected patients (though optimal dose, timing and route of administration are still yet to be confirmed). The most recent meta-analysis (62), including >3,500 women, demonstrated a reduction in risk of preterm birth using vaginal progesterone in high-risk women (RR 0.78). This work suggested that the benefit is most likely in women with a short cervix. The utility among patients with a history of preterm but no concurrent cervical shortening is unclear (63). A recent systematic review and meta-analysis of 87 studies (71 original RCTs and 16 secondary publications with 23,886 women and 32,893 offspring) supported these conclusions noting that in singleton pregnancies progesterone modestly reduced the risk of preterm birth <37 gestational weeks (RR 0.82 [95% CI: 0.71–0.95]) (61).

The use of prophylactic intramuscular progesterone has fallen out of favor over the past several years. The American Food and Drug Administration has withdrawn it from the market in the USA and is not widely utilized in the rest of the world. Cervical cerclage is also efficacious in selected patients. A recent review noted cerclage modestly reduced the risk of preterm birth <37 gestational weeks (RR 0.78 [95% CI 0.69–0.88]) and perinatal mortality (61). While there is debate about the defined population that may benefit from cerclage, it is generally agreed that placement of a cerclage in women who have had previous preterm birth, and who have a short cervix (<25 mm) on transvaginal ultrasonography, may reduce likelihood of subsequent preterm birth. Meta-analysis of four randomized controlled trials (RCTs) including over 600 women showed that in women with a previous second-trimester miscarriage or preterm birth before 37 weeks, cerclage placement significantly reduced delivery before 35 weeks in women with a short cervix [risk ratio (RR) 0.58–0.61] (64). The Arabin Pessary was initially lauded as a minimally invasive means of treating short cervix, but it too has fallen out of favor recently. Trial evidence has been conflicting: a reduction in preterm birth <34 weeks (odds ratio [OR] 0.18), and improved neonatal outcomes were seen in initial trials for women with a short cervix (385 women) (65), though further RCTs and meta-analyses have shown no benefit to cervical pessary in singleton pregnancies (66, 67). The review of 87 studies noted above observed Cervical pessary did not demonstrate any overall effect and low dose aspirin did not affect any outcome, but evidence was based on one underpowered study (61).

There is also growing interest in combined therapy of vaginal progesterone and cerclage or pessary in women at risk of PTB, addressed by two recent meta-analyses. A meta-analysis of 11 studies by Aubin et al., compared cerclage or vaginal progesterone alone versus combined therapy and found significant reduction in PTB and neonatal morbidity with combined therapy when compared to single therapy with either cerclage or vaginal progesterone. Specifically, compared with cerclage only, combined therapy was associated with preterm birth at <34 weeks, <32 weeks, or < 28 weeks, decreased neonatal mortality, increased birthweight, increased gestational age, and a longer interval between intervention and delivery. Compared with progesterone alone, combined therapy was associated with preterm birth at <32 weeks, <28 weeks, decreased neonatal mortality, increased birthweight, and increased gestational age (68). Similarly, Zhuang et al., in a meta-analysis of 16 trials (both cohort studies and randomized controlled trials found 40% reduction in PTB < 34 weeks of gestation, but no significant differences in neonatal outcome for those treated with combined pessary + vaginal progesterone versus vaginal progesterone alone (69). Further prospective studies are needed to identify the obstetrical and cervical characteristics to identify the population most likely to benefit from these combined therapies. Lastly, while initially intended as a treatment for patients at an increased risk of the hypertensive diseases of pregnancy, daily administration of low dose aspirin has also been shown to have modest effect in reducing spontaneous preterm birth in singleton pregnancies in two studies (70, 71). Further large, randomized trials are warranted.

Twins and higher order multiples present difficult challenges in the prevention of preterm birth. In the United States, twins account for 3.1% of all live births, but 20% of all preterm births; overall, 60% of twins are born preterm (<37 weeks of gestation) and 20% are born with early preterm birth (<34 weeks gestation) (72). A recent review of interventions to prevent preterm birth in twin gestations found the use of vaginal progesterone in women with a transvaginal cervical length of <25 mm decreased neonatal morbidity (73). Exam indicated cerclage in patients with cervical dilation of >10 mm showed a significant decrease in preterm birth and associated perinatal mortality (73).

The variability in efficacy seen among these studies could be due to several factors including the population studied, how women at risk were identified, or importantly, interventions started “too late.” A biomarker to identify women at risk prior to events that initiate parturition (e.g., cervical shortening) may allow earlier targeted interventions to prevent preterm birth. It is possible that the multiple pathologies preceding cervical shortening and spontaneous preterm birth, and thus a woman’s response to various preventative interventions, may be differentiated by the expression of different biomarkers. It therefore follows that intervention effects could be more appropriately directed according to early biomarker expressions, i.e., that the underlying pathophysiology may determine the success of one intervention over the other.

## Looking forward

What might be available as novel therapeutic interventions in the near future? There are a variety of prophylactics that are at various stages in the developmental pipeline. For example, to address the association between periodontitis and preterm birth, researchers in Malawi found that daily use of xylitol-containing chewing gum reduced the risk of spontaneous preterm birth and birth weight < 2,500 g (74). While the mechanism is not clear, this would certainly provide an intervention that would be low risk and easy to implement. Silk fibroin based cervical injectables have likewise recently been proposed as a means to augment the cervical tissue stroma among patients with observed cervical shortening (75).

## Conclusion

Reducing preterm birth rates at the global level requires identifying women at risk at a very early stage of pregnancy. Preterm birth can be maternal, fetal, or by risk exposures and pathophysiologic pathways involving both. Besides, fetal and maternal intrauterine organs can independently or synergistically contribute to preterm birth pathways. Identifying the patient at risk (mother, or her fetus or both) and the system that is primarily affected (placenta, fetal membranes, decidua, cervix, myometrium) is extremely important to provide targeted, tissue and biomolecular pathway specific intervention. This can be initiated only with proper diagnosis of the condition through biomarkers of both fetal and maternal origin. Current tests are primarily nonspecific and superficial markers and unlikely to inform to identify the underlying pathophsyiology leading to preterm birth nor identify pathway targeted interventions. Therefore, biomarker discovery, management strategies based on early, mid, and late trimester specific markers have to be developed that identify the underlying cause. The fetus and fetal inflammatory responses are major triggers of preterm labor-initiating mechanisms. However, no tests are currently available to determine the fetus as a patient. Currently, our management strategies are primarily based on maternal clinical indications and interventions to curtail those symptoms are often too late. The contributions from the fetus to the final effector labor pathway have not been explored sufficiently and hence, no biomarker to determine the fetal origin of preterm birth. We propose multi-tier diagnostic strategies based on both maternal and fetal biomarkers. Static risk factors that will not change during the course or pregnancy (e.g., race, ethnicity, genetics, socioeconomic status, environmental factors) and prior history can be used as early markers to categorize subject to high and low risk. This can be followed by dynamic biomarkers in various biological fluids as early as 8 weeks of gestation (e.g., fetal extracellular vesicle based markers in maternal plasma) and create a decision tree to further delineate classification of high risk subjects. This review is not projecting any specific biological specimen, biomarker or approach to detect them, but a generalized strategy to be implemented to address preterm birth syndrome. Due to its complexities and heterogeneities, no single biomarker measured at a given gestational period may indicate risk in a subject. This challenge needs to be addressed systematically through carefully planned biomarker trials. We want to highlight that the problem is not with the biomarkers but identifying them through a well-conducted study.

Currently available tests have good test performance as a “rule-out” test to identify those not at risk for preterm birth. The test performance to identify those at greatest risk is modest and does not identify the majority of patients who will have preterm birth in otherwise low risk populations. Further, most current screening tests fail to identify pathway-specific pathophysiology that would help the provider initiate pathway-specific interventions, frequently resulting empiric intervention and ambiguous results in clinical trials (noted above). Causes of preterm birth are multifactorial, vary by gestational age, and ethnicity and populations that have frequently not been taken into account. Further research into development of pathway-specific biomarkers and pathway-specific intervention is urgently needed to reduce the global burden of preterm birth.

## Author contributions

MG: Conceptualization, Writing – original draft, Writing – review & editing, Project administration. RM: Conceptualization, Writing – review & editing, Writing – original draft. RT: Writing – original draft, Writing – review & editing. NH: Data curation, Writing – original draft, Writing – review & editing. MK: Data curation, Writing – original draft, Writing – review & editing. PS-P: Writing – original draft, Writing – review & editing. BJ: Writing – review & editing. TM: Conceptualization, Writing – original draft, Writing – review & editing.

## Footnotes

^1^https://www.endpreeclampsia.org/
